# Expression of F-actin-capping protein subunit beta, CAPZB, is associated with cell growth and motility in epithelioid sarcoma

**DOI:** 10.1186/s12885-016-2235-z

**Published:** 2016-03-10

**Authors:** Kenta Mukaihara, Yoshiyuki Suehara, Shinji Kohsaka, Daisuke Kubota, Midori Toda-Ishii, Keisuke Akaike, Tsutomu Fujimura, Eisuke Kobayashi, Takashi Yao, Marc Ladanyi, Kazuo Kaneko, Tsuyoshi Saito

**Affiliations:** Department of Orthopedic Surgery, School of Medicine, Juntendo University, Hongo 2-1-1, Bunkyo-ku, Tokyo, 113-8421 Japan; Department of Medical Genomics Graduate School of Medicine, The University of Tokyo, 7-3-1 Hongo, Bunkyo-ku, Tokyo, 113-0033 Japan; Department of Human Pathology, School of Medicine, Juntendo University, Hongo 2-1-1, Bunkyo-ku, Tokyo, 113-8421 Japan; Division of Musculoskeletal Oncology, National Cancer Center Research Institute, 5-1-1 Tsukiji, Chuo-ku, Tokyo, 104-0045 Japan; Laboratory of Biochemical Analysis, Central Laboratory of Medical Sciences, School of Medicine, Juntendo University, Hongo 2-1-1, Bunkyo-ku, Tokyo, 113-8421 Japan; Department of Pathology, Memorial Sloan Kettering Cancer Center, 1275 York Avenue, New York, NY 10065 USA

## Abstract

**Background:**

A previous proteomics study demonstrated the overexpression of F-actin capping protein subunit beta (CAPZB) in tissue specimens of epithelioid sarcoma (EpiS). The aim of the present study was to elucidate the function of CAPZB in EpiS.

**Methods:**

Cellular functional assays were performed in two EpiS cell lines using CAPZB siRNAs. In addition, comparative protein expression analyses using Isobaric Tags for Relative and Absolute Quantitation (i-TRAQ) method were performed to identify the specific proteins whose expression was dysregulated by CAPZB, and analysed the data with the Ingenuity Pathways Analysis (IPA) system using the obtained protein profiles to clarify the functional pathway networks associated with the oncogenic function of CAPZB in EpiS. Additionally, we performed functional assays of the INI1 protein using INI1-overexpressing EpiS cells.

**Results:**

All 15 EpiS cases showed an immunohistochemical expression of CAPZB, and two EpiS cell lines exhibited a strong CAPZB expression. Silencing of CAPZB inhibited the growth, invasion and migration of the EpiS cells. Analysis of protein profiles using the IPA system suggested that SWI/SNF chromatin-remodeling complexes including INI1 may function as a possible upstream regulator of CAPZB. Furthermore, silencing of CAPZB resulted in a decreased expression of INI1 proteins in the INI1-positive EpiS cells, whereas the induction of INI1 in the INI1-deficient EpiS cells resulted in an increased CAPZB mRNA expression.

**Conclusions:**

CAPZB is involved in tumor progression in cases of EpiS, irrespective of the INI1 expression, and may be a potential therapeutic target. The paradoxical relationship between the tumor suppressor INI1 and the oncoprotein CAPZB in the pathogenesis of EpiS remains to be clarified.

**Electronic supplementary material:**

The online version of this article (doi:10.1186/s12885-016-2235-z) contains supplementary material, which is available to authorized users.

## Background

Epithelioid sarcoma (EpiS) is a rare soft tissue sarcoma that affects young adults and is characterized by a tendency toward local recurrence and metastasis [1]. EpiS is classified into two subtypes according the clinicopathological features: a distal form that often arises in the distal extremities as a slow-growing nodule, and a proximal form that tends to arise in deeper areas of the pelvis, perineum and genital tract. Although the clinical course of proximal type may be more aggressive than that of distal type [2, 3], the clinical course is diverse, even for the same subtypes.

Although the molecular pathogenesis of EpiS remains unknown, deletion of the SMARCB1/INI1 tumor-suppressor gene (INI1) was recently reported in cases of proximal-type EpiS [4] and subsequently in cases of distal-type EpiS [5]. Loss of the INI1 expression is observed in approximately 80–90 % of distal and proximal EpiS patients [6, 7], and INI1 genetic inactivation is considered to be responsible for tumorigenesis in cases of EpiS [8]. However, molecular biological aspects related to the progression of EpiS remain unclear, in addition to that associated with INI1, and few functional studies have focused on specific pathways in EpiS cases.

With respect to gaining further insight into the biology of sarcoma, proteomics studies are a powerful approach. Our previous proteomic study demonstrated the CAPZB expression in the tumor tissues of EpiS [9]. In addition, CAPZB is known to increase actin filament depolymerization and capping, which promotes cell motility [10, 11], although functions other than cell motility have not been reported so far. According to the Human Protein Atlas (http://www.proteinatlas.org), CAPZB is also expressed in normal tissue (lymphoid cells, seminiferous ducts, urothelium and placenta exhibited strong staining) and also in certain types of tumors (lymphoma and testicular cancer). In addition, several previous proteomic studies have identified the differential expression of CAPZB [12, 13]. However, the functional roles and clinical impacts of CAPZB expression in these tumors are unknown. Several previous studies have briefly described the functions of CAPZB [11, 14, 15], focusing on its role as a capping protein (CP). CPs are important for the dynamics of actin filament assembly and regulation of the cell shape and movement in vitro [16–19]. However, the functions of CAPZB in EpiS have not yet been elucidated.

In the current study, in order to elucidate the functions of CAPZB in EpiS, we performed functional assays using gene silencing of CAPZB in EpiS cell lines. Consequently, a proteomics study followed by a pathway analysis revealed the SWI/SNF chromatin remodeling complex, which includes INI1, as a possible upstream regulator of CAPZB in the setting of EpiS. We herein describe the oncogenic functions of CAPZB in EpiS, with emphasis on the association with INI1.

## Methods

### Immunohistochemistry

Fifteen cases of EpiS (distal type: 9 cases, proximal type: 6 cases) were chosen from among the pathological records at Juntendo University Hospital or the National cancer Center, Japan According to the World Health Organization (WHO) Classification of Tumors [20], the pathological diagnosis of EpiS for each FFPE case were made by an experienced sarcoma-based pathologist with the conventional immunohistochemical staining such as cytokeratin, EMA, CD34 and vimentin. All cases were positive for CD34, and also positive for at least either cytokeratin or EMA. Loss of INI1 expression was also confirmed for all cases. These fifteen cases of EpiS were used for immunohistochemistry for CAPZB. In brief, 4-μm-thick tissue sections were cut from formalin-fixed, paraffin-embedded blocks. Following deparaffinization, the sections were autoclaved for antigen retrieval in Tris-EDTA buffer (pH 9.0) at 121 °C for 30 min and incubated with a commercial monoclonal antibody against CAPZB (dilution 1: 200, Abcam, ab122980). Immunostaining was carried out according to the streptavidin biotin peroxidase method using a Strept ABC Complex/horseradish peroxidase kit (DAKO, Glostrup, Denmark). Because it has been shown that CAPZB generally localize at the cytoplasm or cellular membrane, we counted only cytoplasmic/membranous staining as positive staining. We uploaded files of the CAPZB immunohistochemical staining of all cases as Additional file 1: Figure S1 and Additional file 2: Figure S2.

Regarding the positive and negative controls of CAPZB IHC, lymphoid cells served as positive control and smooth muscle cells of the vessels as negative control in the immunohistochemical sections, as shown in the Human Protein Atlas homepage (http://www.proteinatlas.org/). Lymphoid cells served as positive control and smooth muscle cells as negative control.

This study was approved by the ethical review board of Juntendo University Hospital and the National Cancer Center, and signed informed consent was obtained from all of the study patients.

### Cell lines

Two EpiS cell lines, VAESBJ (CRL-2138, American Type Culture Collection) and ESX (kindly provided by Sapporo Medical College, Sapporo, Japan) were used in this study. The cells were maintained in DMEM (Life Technologies, Inc., Bethesda, MD) and IMDM (Life Technologies, Inc., Bethesda, MD) supplemented with 10 % FBS, respectively. The cells were incubated at 37 °C in 5 % CO_2_. VAESBJ cells have a deletion of the INI1 gene [8] and show the loss of the INI1 protein expression, whereas ESX cells exhibit the INI1 protein expression [21].

### Knockdown of CAPZB in EpiS cell lines

The EpiS cell lines were treated with 20 nM of two siRNAs for CAPZB (Hs_CAPZB_5777 and Hs_CAPZB_5779, Sigma-Aldrich, St. Louis, MO, USA), siRNA1 (5′ –CUCGUUAGAUUCCUUUCUUTT–3′, antisense 5′ –AAGAAAGGAAUCUAACGAGTT–3′) and siRNA2 (sense 5′ –GGGAUUCCAUCCACGUGGUTT–3′, antisense 5′ –ACCACGUGGAU–GGAAUCCCTT-3′), or siRNA negative control (Sigma-Aldrich, St. Louis, MO, USA) using Lipofectamine™ RNAiMAX reagent (Invitrogen, Carlsbad, CA, USA). At 72 h after transfection, total protein was isolated from each cell line, and the expression level of CAPZB was validated using a Western blotting analysis.

### Western blotting

The proteins were separated via SDS-PAGE and transferred to nitrocellulose membranes. The membranes were incubated with either of the following antibodies: mouse monoclonal antibodies against CAPZB (dilution 1: 1,000, Abcam, ab122980), INI1 (dilution 1: 500, BD Transduction Laboratories, 612110) or GAPDH (dilution 1: 500, Santa Cruz, sc-32233). After incubation, the membranes were washed three times with Tris-EDTA buffer and then reacted with horseradish peroxidase-conjugated secondary antibodies (1:1,000 dilution, GE Healthcare Biosciences).

### Preparation of retrovirus and transduction of the cell lines

The Tet-On expression system was used for transduction of the genes of interest, and the TRMPV-Neo (Addgene Plasmid #27990) vector system was used for retrovirus production (PMID: 21131983). The sh-RNA site was deleted from the TRMPV-Neo vector for the DsRed overexpression vector (TRMPV-DsRed-Neo), and SMARCB1 was subcloned from human diploid fibroblasts into the position of DsRed for TRMPV-SMARCB1-Neo. MSCV-rtTA-EcoR-Puro was kindly provided by Dr. Scott Lowe. Retroviruses were obtained using 293 T cells as packaging cells, infected into the EpiS lines and selected with 4 μg/ml of puromycin or 500 μg/ml of G418 (Invitrogen, Carlsbad, CA, USA). Transcription of the TRE-regulated target gene was stimulated by rtTA in the presence of 10 ng/ml of doxycycline (Dox) (Sigma-Aldrich, St. Louis, MO, USA).

### Cell proliferation assay

VAESBJ and ESX cells were seeded in 96-well plates at 2,000 cells/well and 3,000 cells/well, respectively on day 1. On day 1, transfection was performed with 20 nM of the same siRNA reagents described above. Cell proliferation was monitored using the Cell Counting Kit-8 (Dojindo, Kumamoto, Japan) and a microplate reader to measure the absorbance of the culture medium at 450 nm according to the manufacturer’s instruction manual. All proliferation experiments were performed in triplicate, and the results were averaged.

### Invasion assay

The invasion assays were performed using 24-well BD BioCoat Matrigel Invasion Chambers (BD Biosciences, Franklin Lakes, NY), according to the manufacturer’s protocol. VAESBJ and ESX cell suspensions were prepared at a density of 8 × 10^4^ cells/mL and 3 × 10^5^ cells/mL in 0.5-ml serum-free medium and added to the gel chamber insert. After 48 h of incubation, non-invading cells were removed with cotton swabs, and invading cells were stained using Diff-Quick reagent (Sysmex, Kobe, Hyogo, Japan). The number of invading cells was counted, and the invasion index was calculated as the percent invasion of transfected cells/non-transfected cells.

### Scratch assay

The rate of cell migration was assessed using a scratch assay. The VAESBJ cell line transfected with CAPZB siRNA was seeded on a 6-well plate and allowed to reach confluence. After scratching the bottom of the well with a pipette tip, the monolayer of cells was washed with PBS to remove detached cells. The remaining adherent cells were incubated in medium containing 0.2 % FBS, and the area of the scratch wound was evaluated at 48 h after transfection. The experiments were performed in triplicate.

### Proteomic analysis using iTRAQ (isobaric tags for relative and absolute quantification) and mass spectrometry

Isobaric tags for relative and absolute quantification (iTRAQ), a form of chemical labeling mass spectrometry, were created according to the company’s protocol [22]. Briefly, the cell lysate was extracted from each cell and subjected to a LC-shot gun analysis using the iTRAQ method, as previously described [23]. Prior to the iTRAQ analysis, the lysate samples were concentrated and buffer exchanged using a 3.5-kDa molecular weight cut-off spin concentrator (TOMY SEIKO CO., LTD, Tokyo, Japan) then digested for 24 h with 10 μg of L-1-(4-tosylamido)-2-phenylethyl tosyl phenylalanyl chloromethyl ketone (TPCK)-treated trypsin. Each peptide solution was labelled with one of the four iTRAQ reagents (iTRAQ reporter ions of 114, 115, 116 and 117 mass/charge ratio) according to the manufacturer’s protocol (AB SCIEX, Framingham, MA, USA). The labelled peptides were pooled and fractionated via strong cation exchange (SCX) using a ChromXP C18-CL column (Eksigent parts of AB SCIEX, Dublin, California, USA) and analyzed with nano LC-MS/MS [24]; nano LC-MS/MS was performed using a TripleTOF® 5600 mass spectrometer for MS/MS (AB SCIEX) interfaced with a nano LC system (Eksigent parts of AB SCIEX).

### Peptide identification

Protein identification and relative quantification were carried out as previously described (ProteinPilot™ Software Version 4.5) [25]. Functional definitions of the variable protein contents were searched against the Swissport database (Release, 6/20/2014) using the search algorithm contained within the ProteinPilot™ Software and Analyst® TF Software programs (AB SCIEX). The protein ratios were normalized using the overall median ratio for all peptides in the sample for the separate ratios in each individual experiment. A confidence cutoff value for protein identification of >95 % was applied for protein identification, and a >1.2-fold change cutoff was selected for all of the iTRAQ ratios in order to classify proteins as upregulated or downregulated as previously described [26].

### Pathway analysis

The Ingenuity Pathways Analysis (IPA) (Ingenuity Systems, Redwood City, CA) software program was further used to determine the functional pathways represented by the identified genes. The IPA software package contains a database of biological interactions among genes and proteins, which was used to calculate the probability of a relationship between each canonical pathway, upstream pathway and the identified proteins. The IPA program scans the set of input proteins to identify networks using the Ingenuity Pathway Knowledge Base (IPKB) for interactions between identified proteins and known and hypothetical interacting genes stored in the IPA software database.

### Statistical Analysis

Data from the western blotting and quantitative-PCR were analyzed using the t-test. The significance of differences in the functional assays of EpiS cell lines following transfection was evaluated using the t-test.

The statistical analysis of protein expression profiles is based on *p*-value and threshold using Protein Pilot version 4.5 software (AB SCIEX) [27].

## Results

### CAPZB expression in EpiS clinical samples and EpiS cells

**Fig. 1:**
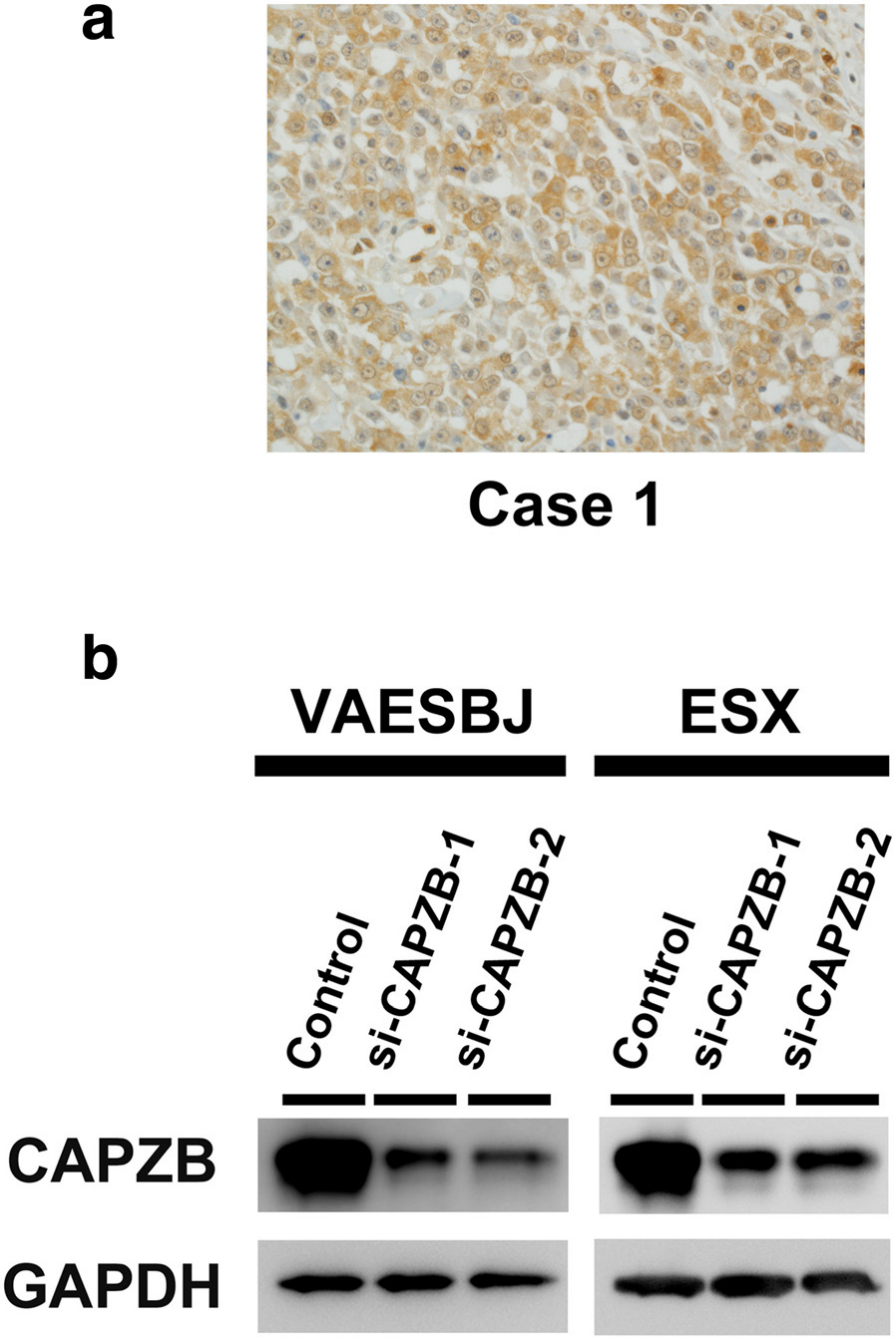
Localization and expression of CAPZB in EpiS. **a** Immunohistochemistry in representative EpiS case shows the CAPZB expression in the tumor cells. **b** CAPZB is strongly expressed in the two EpiS cell lines. Treatment with the two siRNAs (si-CAPZB-1 and si-CAPZB-2) significantly decreases the expression levels of CAPZB in the two cell lines

Fifteen cases of EpiS were examined using immunohistochemistry to confirm the protein expression and localization of CAPZB. In all 15 cases, more than 70 ~ 80 % of tumor cells were positive for CAPZB and CAPZB was localized in the cytoplasm. Some cases also showed nuclear staining (Fig. 1a, Additional file 1: Figure S1 and Additional file 2: Figure S2). These results were consistent with the findings of our previous proteomic analysis showing that tumor tissues of ES have higher CAPZB expression levels than normal tissues [9]. In the present study, we also confirmed the expressions of CAPZB in the two EpiS cell lines (VAESBJ and ESX cells) using WB (Fig. 1b).

### Functional analysis of CAPZB

**Fig. 2:**
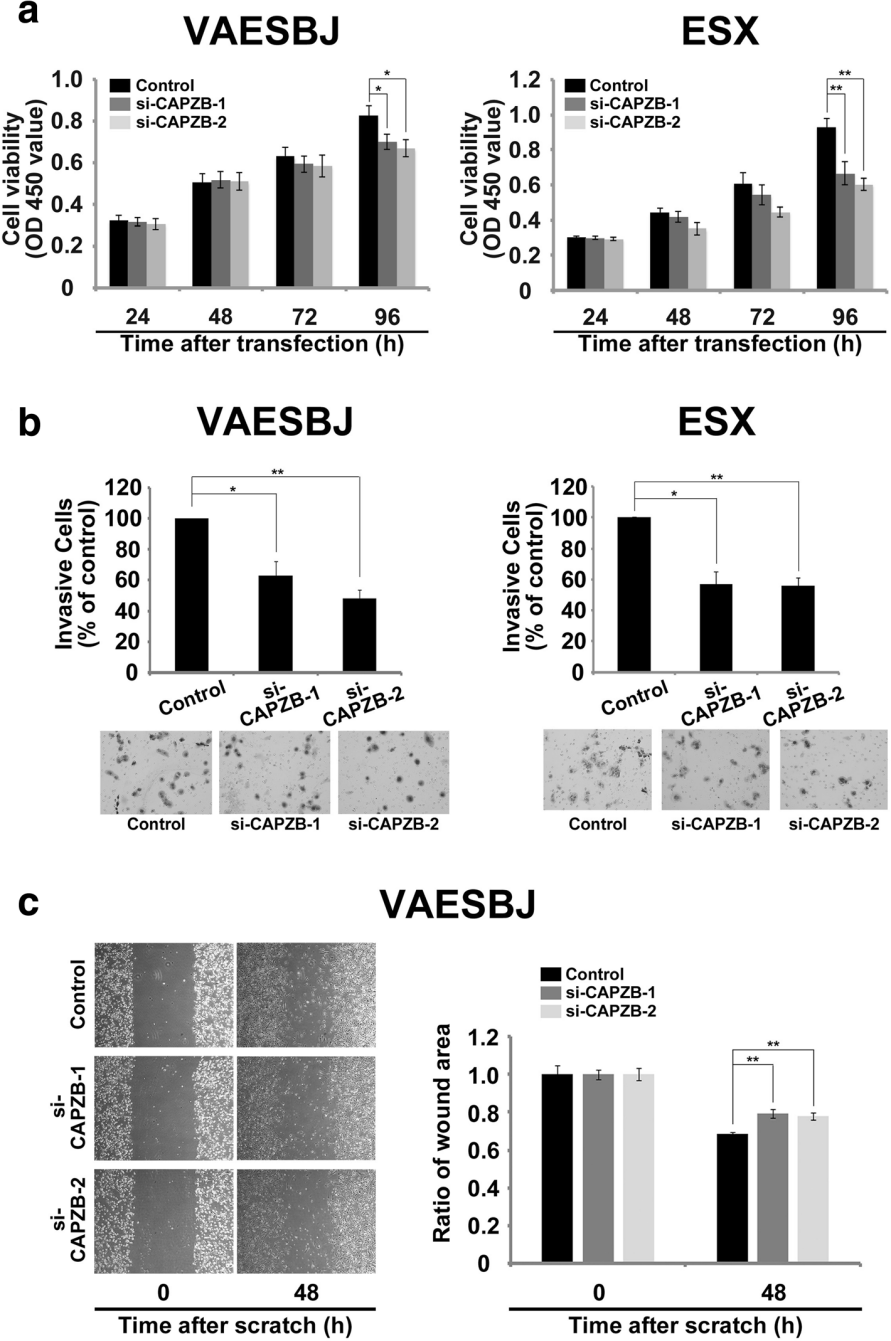
Progressive effect of CAPZB in EpiS cellular function. **a** In the proliferation assays, si-CAPZB-1 and si-CAPZB-2 inhibited cell growth by 85 % and 81 % in the VAESBJ cells and 71 % and 65 % in the ESX cells, respectively (*p* < 0.01, *p* < 0.05, Figure 2B left and right, respectively). **b** In the invasion assays, si-CAPZB-1 and si-CAPZB-2 decreased cell invasion by 63 % and 48 % in the VAESBJ cells and 57 % and 56 % in the ESX cells, respectively (*p* < 0.01, *p* < 0.05, Figure 2c left and right, respectively). **c** In the scratch assays, CAPZB silencing suppressed the area of wound healing in the VAESBJ cells (*p* < 0.01, Control: 32 %, si-CAPZB-1: 21 %, si-CAPZB-2: 22 %, Fig. 2d)

In order to investigate the cellular functions of CAPZB in EpiS, we performed siRNA assays of CAPZB using the two EpiS cell lines. As a result, treatment with two siRNAs (si-CAPZB-1 and si-CAPZB-2) significantly decreased the expression levels of CAPZB compared to that observed in the control cells for both the VAESBJ and ESX cells (Fig. 1b). Following gene silencing of CAPZB by the two siRNAs in both EpiS cell lines, we performed in vitro assays consisting of cell proliferation, invasion and scratch assays. In the cell proliferation assays, knockdown of CAPZB significantly decreased the rate of cell growth at 96 h after transfection in both the VAESBJ and ESX cells (Fig. 2a). In the invasion assays, the CAPZB-silenced cells demonstrated markedly decreased cell invasion (Fig. 2b). In the scratch assays, silencing of CAPZB in the VAESBJ cells significantly suppressed cell migration compared to that observed in the control cells (Fig. 2c). We were unable to obtain cell migration data for the ESX cells because these cells easily detached from the culture plate with scratching.

Next, we performed a proteomics approach using i-TRAQ assays with the CAPZB siRNA-transfected EpiS cells in order to determine the differences in the protein expression profile according to the knockdown of CAPZB in EpiS. Consequently, the protein expression profiles differed significantly between the CAPZB-silenced cells and the control cells (*p* < 0.05). This analysis revealed protein profiles consisting of 26 downregulated proteins and 39 upregulated proteins in the VAESBJ cells (*p* < 0.05, Table 1), as well as 69 downregulated proteins and 41 upregulated proteins in the ESX cells (*p* < 0.05, Table 2). The numbers of commonly upregulated and downregulated proteins between two cell lines were 3 and 8, respectively. In order to further understand the biological networks associated with CAPZB, we employed a network analysis using the IPA system with the above obtained protein profiles in the VAESBJ and ESX cells. The results of the IPA analyses are shown in Additional file 1: Figure S1, Additional file 2: Figure S2, Additional file 3: Table S1 and Additional file 4: Table S2. These results pointed to several SWI/SNF chromatin-remodeling complexes as possible upstream regulators or critical pathways among the identified network lists. We also found INI1 to be included in the identified SWI/SNF chromatin-remodeling complex list (Additional file 3: Table S1 and Additional file 4: Table S2).

**Table 1:** Protein profiles of CAPZB-regulated proteins in the VAESBJ cells

^a^Accession no.	Symbol	Protein name	Fold difference	*P* value
P07355	ANXA2	Annexin A2	1.90	0.00E+00
P02768	ALBU	Serum albumin	1.85	2.17E-02
P08195	4 F2	Isoform 3 of 4 F2 cell-surface antigen heavy chain	1.63	1.00E-04
Q16222	UAP1	UDP-N-acetylhexosamine pyrophosphorylase	1.55	3.10E-03
Q09666	AHNK	Neuroblast differentiation-associated protein AHNAK	1.53	0.00E+00
P08243	ASNS	Asparagine synthetase [glutamine-hydrolyzing]	1.45	2.00E-04
Q96HC4	PDLI5	PDZ and LIM domain protein 5	1.44	1.37E-02
P13797	PLST	Plastin-3	1.44	0.00E+00
P21589	5NTD	5′-nucleotidase	1.43	5.10E-03
P15144	AMPN	Aminopeptidase N	1.42	1.00E-03
P26639	SYTC	Threonine—tRNA ligase, cytoplasmic	1.39	2.00E-04
Q05682	CALD1	Isoform 5 of Caldesmon	1.38	3.20E-03
P06756	ITAV	Integrin alpha-V	1.33	2.14E-02
P21333	FLNA	Isoform 2 of Filamin-A	1.32	0.00E+00
P05556	ITB1	Integrin beta-1	1.32	0.00E+00
P18206	VINC	Vinculin	1.31	0.00E+00
P06737	PYGL	Glycogen phosphorylase, liver form	1.30	1.13E-02
O43175	SERA	D-3-phosphoglycerate dehydrogenase	1.30	1.27E-02
P00390	GSHR	Glutathione reductase, mitochondrial	1.29	3.08E-02
P16989	YB0X3	Y-box-binding protein 3	1.29	3.19E-02
Q9UKK3	PARP4	Poly [ADP-ribose] polymerase 4	1.29	4.22E-02
Q9Y617	SERC	Phosphoserine aminotransferase	1.28	1.80E-02
P06733	ENOA	Alpha-enolase	1.26	1.00E-03
P00352	AL1A1	Retinal dehydrogenase 1	1.26	1.00E-04
P49591	SYSC	Serine—tRNA ligase, cytoplasmic	1.24	9.70E-03
P20810	ICAL	Isoform 9 of Calpastatin	1.23	2.19E-02
P41250	SYG	Glycine—tRNA ligase	1.23	2.51 E-02
O60884	DNJA2	DnaJ homolog subfamily A member 2	1.21	2.44E-02
Q9NSE4	SYIM	Isoleucine—tRNA ligase, mitochondrial	1.21	2.46E-02
P61289	PSME3	Proteasome activator complex subunit 3	1.21	1.26E-02
P27824	CALX	Calnexin	1.20	1.24E-02
P15311	EZRI	Ezrin	1.20	2.00E-04
Q9BXJ9	NAA15	N-alpha-acetyltransferase 15, NatA auxiliary subunit	1.19	3.35E-02
P43490	NAMPT	Nicotinamide phosphoribosyltransferase	1.18	1.59E-02
Q01813	K6PP	6-phosphofructokinase type C	1.18	2.47E-02
O75369	FLNB	Isoform 2 of Filamin-B	1.16	6.00E-04
P22102	PUR2	Trifunctional purine biosynthetic protein adenosine-3	1.15	5.80E-03
P49411	EFTU	Elongation factor Tu, mitochondrial	1.10	1.77E-02
P38646	GRP75	Stress-70 protein, mitochondrial	1.10	4.13E-02
Q00610	CLH1	Clathrin heavy chain 1	0.91	4.92E-02
P13010	XRCC5	X-ray repair cross-complementing protein 5	0.90	2.90E-02
P25685	DNJB1	DnaJ homolog subfamily B member 1	0.85	1.49E-02
P61981	1433G	14-3-3 protein gamma	0.85	2.42E-02
P30086	PEBP1	Phosphatidylethanolamine-binding protein 1	0.84	1.79E-02
P09382	LEG1	Galectin-1	0.84	3.48E-02
P08133	ANXA6	Annexin A6	0.84	2.00E-04
043747	AP1G1	Isoform 2 of AP-1 complex subunit gamma-1	0.83	2.44E-02
P05787	K2C8	Keratin, type II cytoskeletal 8	0.82	4.00E-04
P10644	KAPO	cAMP-dependent protein kinase type I-alpha regulatory subunit	0.81	4.19E-02
Q9NZ01	TECR	Very-long-chain enoyl-CoA reductase	0.81	4.10E-02
Q9BUJ2	HNRL1	Heterogeneous nuclear ribonucleoprotein U-like protein 1	0.81	3.70E-02
O14579	COPE	Coatomer subunit epsilon	0.80	4.18E-02
P08727	K1C19	Keratin, type I cytoskeletal 19	0.79	4.10E-03
P07108	ACBP	Isoform 3 of Acyl-CoA-binding protein	0.79	2.54E-02
P63104	1433Z	14-3-3 protein zeta/delta	0.78	1.61 E-02
Q15274	NADC	Nicotinate-nucleotide pyrophosphorylase [carboxylating]	0.78	4.40E-02
P25705	ATPA	ATP synthase subunit alpha, mitochondrial	0.77	1.82E-02
P04080	CYTB	Cystatin-B	0.77	1.03E-02
P53634	CATC	Dipeptidyl peptidase 1	0.77	3.85E-02
Q07955	SRSF1	Serine/arginine-rich splicing factor 1	0.76	1.99E-02
Q9NQC3	RTN4	Isoform 2 of Reticulon-4	0.71	2.76E-02
P00568	KAD1	Adenylate kinase isoenzyme 1	0.70	5.10E-03
Q7L1QB	BZW1	Basic leucine zipper and W2 domain-containing protein 1	0.65	1.10E-03
Q9ULV4	COR1C	Isoform 3 of Coronin-1C	0.63	2.10E-03
P52907	CAZA1	F-actin-capping protein subunit alpha-1	0.62	1.71E-02
P04264	K2C1	Keratin, type II cytoskeletal 1	0.39	2.00E-04
P04179	SODM	Superoxide dismutase [Mn], mitochondrial	0.34	3.08E-02
P35527	K1C9	Keratin, type I cytoskeletal 9	0.29	4.20E-03

^a^Accession numbers of proteins were derived from Swiss-Plot data base

**Table 2:** Protein profiles of CAPZB-regulated proteins in the ESX cells

^a^Accession no.	Symbol	Name	Fold difference	*P* value
Q15738	NSDHL	Sterol-4-alpha-carboxylate 3-dehydrogenase, decarboxylating	2.02	2.50E-03
Q9P287	BCCIP	BRCA2 and CDKN1 A-interacting protein	2.02	3.48E-02
Q9NZL4	HPBP1	Hsp70-binding protein 1	1.90	2.65E-02
P14174	MIF	Macrophage migration inhibitory factor	1.84	3.30E-02
P20591	MX1	Interferon-induced GTP-binding protein Mx1	1.82	2.64E-02
P20936	RASA1	Ras GTPase-activating protein 1	1.76	4.03E-02
O95373	IP07	Importin-7	1.73	7.70E-03
P27144	KAD4	Adenylate kinase 4, mitochondrial	1.67	1.44E-02
Q96EK5	KBP	KIF1—binding protein	1.65	1.70E-02
Q9P2J5	SYLC	Leucine—tRNA ligase, cytoplasmic	1.64	1.17E-02
P49354	FNTA	Protein farnesyltransferase/geranylgeranyltransferase type-1 subunit alpha	1.63	7.40E-03
P25786	PSA1	Proteasome subunit alpha type-1	1.58	3.89E-02
P49773	HINT1	Histidine triad nucleotide-binding protein 1	1.52	7.20E-03
P51003	PAPOA	Poly(A) polymerase alpha	1.51	2.49E-02
P09211	GSTP1	Glutathione S-transferase P	1.50	4.95E-02
P07339	CATD	Cathepsin D	1.50	2.52E-02
P12277	KCRB	Creatine kinase B-type	1.46	4.14E-02
P61289	PSME3	Proteasome activator complex subunit 3	1.44	4.77E-02
Q4J6C6	PPCEL	Prolyl endopeptidase-like	1.43	4.89E-02
Q6IBS0	TWF2	Twinfilin-2	1.43	4.96E-02
P14625	ENPL	Endoplasmin	1.42	0.00E+00
P55060	XP02	Exportin-2	1.42	1.30E-03
P08238	HS90B	Heat shock protein HSP 90-beta	1.39	4.10E-02
P30041	PRDX6	Peroxiredoxin-6	1.38	1.02E-02
O00410	IP05	Isoform 3 of Importin-5	1.37	2.24E-02
Q14697	GANAB	Neutral alpha-glucosidase AB	1.36	2.03E-02
P13797	PLST	Plastin-3	1.36	5.50E-03
043681	ASNA	ATPase ASNA1	1.32	3.37E-02
P27824	CALX	Calnexin	1.31	3.94E-02
P48735	IDHP	Isocitrate dehydrogenase [NADP], mitochondrial	1.30	4.29E-02
P50395	GDIB	Rab GDP dissociation inhibitor beta	1.30	2.38E-02
O60488	ACSL4	Long-chain-fatty-acid—CoA ligase 4	1.29	1.79E-02
P80303	NUCB2	Nucleobindin-2	1.29	2.91 E-02
P05455	LA	Lupus La protein	1.27	2.40E-02
Q15084	PDIA6	Protein disulfide-isomerase A6	1.26	4.60E-03
Q16576	RBBP7	Histone-binding protein RBBP7	1.25	4.35E-02
Q99832	TCPH	T-complex protein 1 subunit eta	1.24	4.78E-02
P50990	TCPQ	T-complex protein 1 subunit theta	1.21	9.70E-03
P10809	CH60	60 kDa heat shock protein, mitochondrial	1.18	4.64E-02
Q06830	PRDX1	Peroxiredoxin-1	1.18	2.23E-02
P50502	F10A1	Hsc70-interacting protein	1.15	1.39E-02
O75369	FLNB	Isoform 2 of Filamin-B	0.85	1.33E-02
P12814	ACTN1	Isoform 3 of Alpha-actinin-1	0.84	3.51 E-02
P35580	MYH10	Myosin-10	0.84	1.10E-03
P09496	CLCA	Clathrin light chain A	0.84	4.71 E-02
P21333	FLNA	Isoform 2 of Filamin-A	0.84	5.00E-04
P26583	HMGB2	High mobility group protein B2	0.83	1.86E-02
O60841	IF2P	Eukaryotic translation initiation factor 5B	0.83	3.66E-02
Q08257	QOR	Quinone oxidoreductase	0.83	2.95E-02
Q9Y3A5	SBDS	Ribosome maturation protein SBDS	0.82	4.20E-02
Q14203	DCTN1	Dynactin subunit 1	0.81	4.67E-02
Q00839	HNRPU	Heterogeneous nuclear ribonucleoprotein U	0.81	2.61 E-02
O15371	EIF3D	Eukaryotic translation initiation factor 3 subunit D	0.79	4.68E-02
Q6UB35	C1TM	Monofunctional C1~tetrahydrofolate synthase, mitochondrial	0.79	3.39E-02
P39019	RS19	40S ribosomal protein S19	0.77	1.28E-02
P27816	MAP4	Isoform 6 of Microtubule-associated protein 4	0.77	4.66E-02
Q00688	FKBP3	Peptidyl-prolyl cis-trans isomerase FKBP3	0.77	3.16E-02
Q9BUJ2	HNRL1	Heterogeneous nuclear ribonucleoprotein U—like protein 1	0.76	1.06E-02
P08195	4 F2	Isoform 3 of 4 F2 cell-surface antigen heavy chain	0.76	2.10E-02
P52272	HNRPM	Heterogeneous nuclear ribonucleoprotein M	0.70	2.82E-02
Q15274	NADC	Nicotinate-nucleotide pyrophosphorylase [carboxylating]	0.76	3.26E-02
Q12906	ILF3	Interleukin enhancer-binding factor 3	0.70	2.04E-02
P08670	VIME	Vimentin	0.75	1.00E-04
P18124	RL7	0OS ribosomal protein L7	0.74	1.15E-02
Q92841	DDX17	Isoform 4 of Probable ATP-dependent RNA helicase DDX17	0.74	5.60E-03
P62906	RL10A	80S ribosomal protein L10a	0.73	2.09E-02
P16989	YBOX3	Y-box-binding protein 3	0.73	1.50E-02
P05161	ISG15	Ubiquitin—like protein ISG15	0.73	2.87E-02
P06748	NPM	Nucleophosmin	0.72	1.07E-02
O00571	DDX3X	ATP-dependent RNA helicase DDX3X	0.71	7.00E-03
P62424	RL7A	80S ribosomal protein L7a	0.71	3.87E-02
P46776	RL27A	60S ribosomal protein L27a	0.71	3.40E-02
P83881	RL30A	60S ribosomal protein L36a	0.70	2.03E-02
P46777	RL5	60S ribosomal protein L5	0.70	2.00E-04
P62913	RL11	60S ribosomal protein L11	0.70	1.04E-02
P50914	RL14	60S ribosomal protein L14	0.70	2.61 E-02
P62280	RS11	40S ribosomal protein S11	0.70	1.18E-02
P62917	RL8	60S ribosomal protein L8	0.70	5.00E-03
P84098	RL19	60S ribosomal protein L19	0.69	1.27E-02
P62241	RS8	40S ribosomal protein S8	0.69	2.37E-02
P6322O	RS21	40S ribosomal protein S21	0.69	1.24E-02
Q05682	CALD1	Isoform 5 of Caldesmon	0.69	1.00E-04
P54886	P5CS	Delta-1 -pyrroline-5-carboxylate synthase	0.68	1.10E-03
P01247	RS3A	40S ribosomal protein S3a	0.68	7.00E-04
P35613	BASI	Isoform 2 of Basigin	0.68	2.83E-02
P26373	RL13	60S ribosomal protein L13	0.68	2.19E-02
Q14789	GOGB1	Golgin subfamily B member 1	0.67	2.56E-02
P05023	AT1A1	Sodium/potassium-transporting ATPase subunit alpha-1	0.67	2.00E-04
P52907	CAZA1	F-actin-capping protein subunit alpha-1	0.66	2.86E-02
P09382	LEG1	Galectin-1	0.66	1.40E-02
P09874	PARP1	Poly [ADP-ribose] polymerase 1	0.66	2.00E-04
P6275O	RL23A	60S ribosomal protein L23a	0.66	4.00E-04
Q8NC51	PAIRB	Plasminogen activator inhibitor 1 RNA-binding protein	0.65	1.00E-04
P46779	RL28	60S ribosomal protein L28	0.65	1.13E-02
P49748	ACADV	Very long-chain specific acyl-CoA dehydrogenase, mitochondrial	0.65	1.74E-02
Q01082	SPTB2	Spectrin beta chain, non-erythrocytic 1	0.64	0.00E+00
Q07955	SRSF1	Serine/arginine-rich splicing factor 1	0.64	3.94E-02
Q9NZI8	IF2B1	Insulin-like growth factor 2 mRNA-binding protein 1	0.63	4.10E-03
Q02878	RL6	60S ribosomal protein L6	0.62	2.90E-03
Q15233	NONO	Non-POU domain-containing octamer-binding protein	0.62	2.64E-02
P07910	HNRPC	Heterogeneous nuclear ribonucleoproteins C1/C2	0.61	0.00E+00
P08133	ANXA6	Annexin A6	0.60	0.00E+00
Q07020	RL18	60S ribosomal protein L18	0.60	1.00E-02
Q7L1Q6	BZW1	Basic leucine zipper and W2 domain-containing protein 1	0.59	1.00E-04
Q16643	DREB	Drebrin	0.58	1.00E-04
Q9ULV4	COR1C	Isoform 3 of Coronin-1C	0.57	6.30E-03
P47756	CAPZB	Isoform 2 of F-actin-capping protein subunit beta	0.55	3.01 E-02
043707	ACTN4	Alpha-actinin-4	0.54	0.00E+00
P16070	CD44	Isoform 11 of CD44 antigen	0.54	3.10E-03
P22626	ROA2	Heterogeneous nuclear ribonucleoproteins A2/B1	0.52	2.12E-02

^a^Accession numbers of proteins were derived from Swiss-Plot data base

**Fig. 3:**
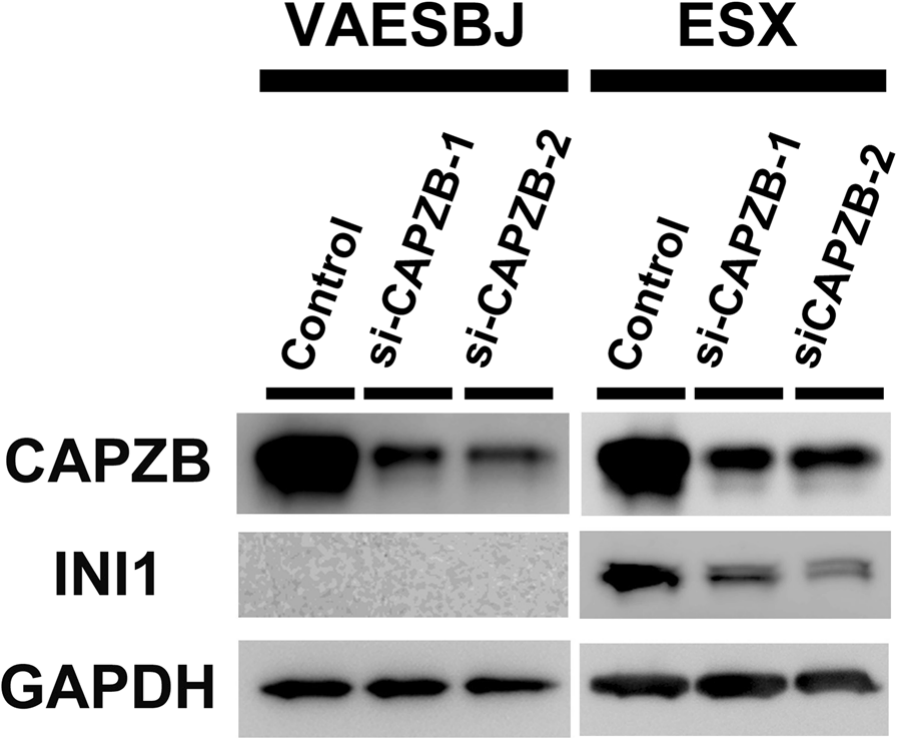
Association between the expression of CAPZB and INI1 in EpiS cells. In the ESX cells, suppression of the CAPZB expression inhibited the expression of INI1 in both siRNA assays. We were unable to evaluate the association between CAPZB and INI1 in the VAESBJ cells, because this cell line had deletions of INI1 and did not show an expression of INI1

In order to elucidate the possible association between the INI1 and CAPZB expression, we performed siRNA assays of CAPZB in the two EpiS cell lines and measured the expression levels of CAPZB and INI1 using WB. In the ESX cell line (without the loss of INI1), gene silencing of CAPZB led to a decrease in the expression level of INI1 (Fig. 3, right). In the VAESBJ cell line (with the loss of INI1), the INI1 expression remained lost (Fig. 3, left).

**Fig. 4:**
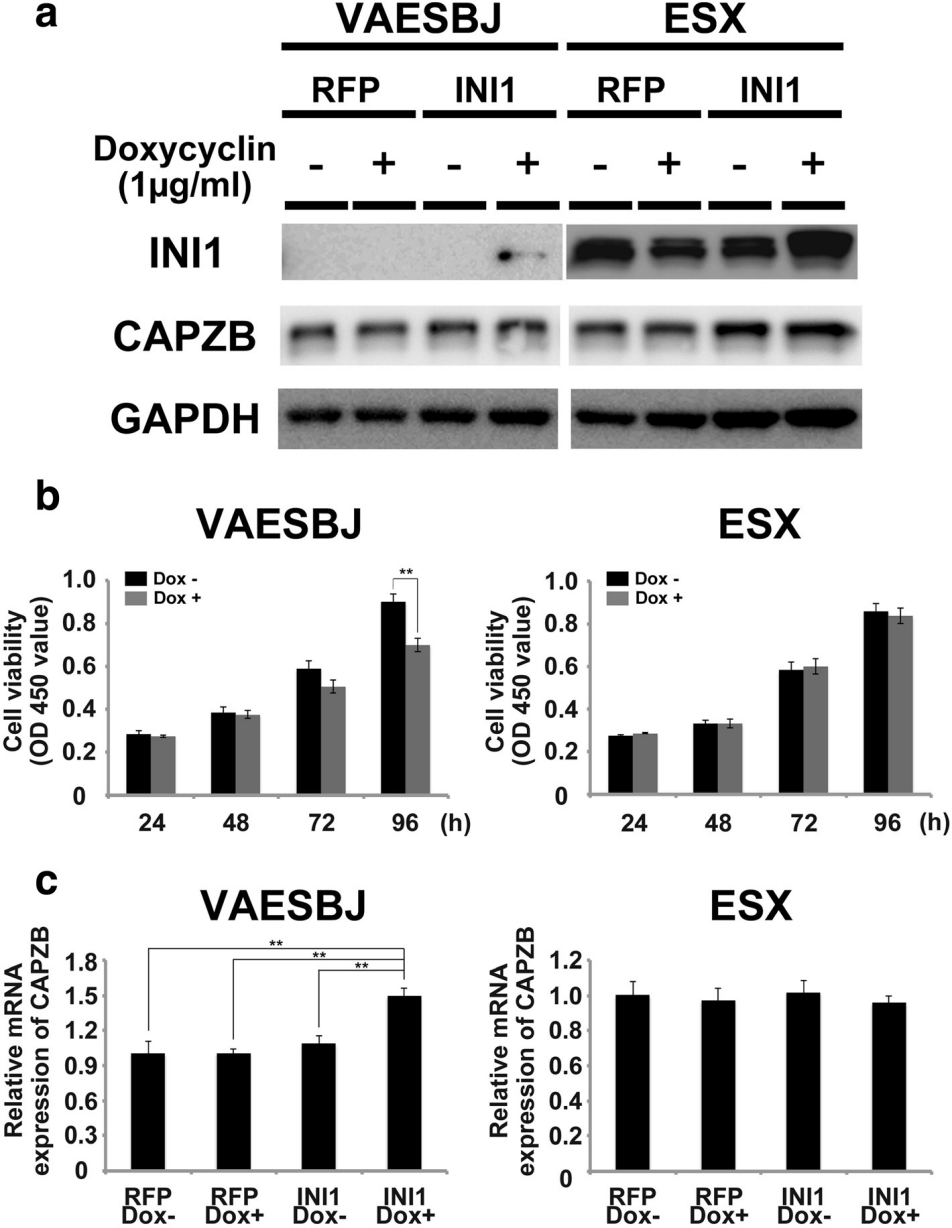
The expression levels of CAPZB and cell growth in INI1-overexpressing EpiS cells. **a** In the VAESBJ cell line, the INI1 expression was induced in the doxycycline (Dox)-induced INI1-overexpressing cells, whereas the other three cells did not show an expression of INI1 (**a**, left panel). In the ESX cell line, all four cells expressed INI1, and the Dox-induced INI1-overexpressing cells had higher expression levels than the other three cells (**a**, right panel). Regarding the expression levels of CAPZB, the WB assay demonstrated no remarkable differences among the four types of cells for both the VAESBJ and ESX cells. **b** In the cell proliferation assay, a significant growth inhibition was observed in the Dox-induced INI1-overexpressing cells (*p* < 0.01, B, left panel). The cell proliferation assays of the ESX cells showed no significant differences in growth between the INI1-overexpressing cells and the control cells (**b**, right panel). **c** Relative expression of CAPZB mRNA in the VAESBJ and ESX cells (right and left panel, respectively). The mean expression levels of CAPZB in the RFP DOX-, RFP DOX+, INI1 Dox- and INI1 Dox + cells of each cell line, as determined using real-time PCR are shown, normalized to the expression of CAPZB mRNA in the RFP DOX- cells. In the VAESBJ cells, the INI1 Dox + cells had higher mRNA expression levels of CAPZB than the other three cells (**c**, left panel). On the other hand, in the ESX cells, there were no significant differences in the mRNA expression levels of CAPZB among the four cells (**c**, right panel)

Next, we attempted to assess the effects of INI1 overexpression in the VAESBJ and ESX cells. These EpiS cell lines were stably transfected with either Dox-inducible empty (RFP) or the INI1 expression vector, and the expression levels of INI1 were confirmed with WB. In the VAESBJ cell line, in which INI1 was lost, Dox induction in the INI1-transfected cells (INI1+) induced the INI1 expression (Fig. 4a, upper left). In the ESX cell line, Dox induction in the INI1-transfected ESX cells resulted in a higher expression of INI1 than that noted in the other three cell lines (Fig. 4a, upper right). According to the presence of INI1 induction, a real-time PCR assay revealed that the expression level of CAPZB in the Dox-induced INI1-transfected cells was significantly higher than that observed in the other three cells among the VAESBJ cells (Fig. 4c). However, the WB assay did not detect apparent differences in the CAPZB expression (Fig. 4a, middle). Based on these findings, it was difficult to assume the direct effect of INI1 on CAPZB in EpiS cells. Regarding the proliferation of the Dox-induced INI1-overexpressing cells, the cell growth of the Dox-induced INI1-overexpressing VAESBJ cells was markedly suppressed compared to that of the control cells (Fig. 4b, left). However, the Dox-induced INI1-overexpression in VAESBJ cells did not affect the migration and invasion properties (Data not shown). In contrast, in the ESX cell lines, Dox-induced INI1-overexpression did not affect cell proliferation (Fig. 4b, right).

## Discussion

Capping proteins are known to increase actin filament depolymerization and promote cell motility [10, 11]. However, the associations between the expression levels of capping proteins, including CAPZB, and cancer phenotypes remain unknown. Furthermore, although we have previously reported high CAPZB expression in the tumor tissues of EpiS [9], relative expression of CAPZB in EpiS compared to other types of tumors is unknown, therefore it is difficult to refer to the association between the relative expression level of CAPZB and aggressive behavior of the tumors. Recently, CAPZA1, a member of the capping protein family, was reported to have prognostic value and a suppressive effect on cell migration and invasion in gastric cancer tissue [28], whereas the overexpression of actin-capping proteins has been shown to modulate cell motility in vitro, suggesting their potentially important role in promoting cell motility in the setting of pancreatic cancer [29]. These findings indicate that members of the capping protein family, including CAPZB, contribute to tumor progression in several cancers in a tumor-specific manner. In the present study, we confirmed the CAPZB expression in EpiS clinical samples and cell lines and showed that CAPZB contributes to tumor progression in the setting of EpiS by promoting cellular proliferation, invasion and migration, thus demonstrating that CAPZB functions as an oncoprotein in the pathogenesis of EpiS.

It is also of interest to identify which biological pathways are involved in promoting the tumor progression induced by CAPZB in cases of EpiS. Therefore, we performed a proteomics study to examine changes in the protein expression profiles according to the knockdown of CAPZB. Protein profiles differentially expressed based on the knockdown of CAPZB included MX1 and BCCIP as upregulated proteins and CD44 and FLNB as downregulated proteins. Some of these proteins were identified as down stream regulator of SWI/SNF complexes including INI1 by using IPA analysis (Table S1 and S2). MX1, Interferon-induced GTP-binding protein MX1, was involved in antiviral responses [30, 31]. Previous proteomic study referred this protein as to be a marker of lymph node metastasis and having functional role of tumor invasion in colorectal carcinoma [32]. BCCIP, protein which interacts with BRCA2 and CDKN1A, has been implicated in many cellular processes including cell cycle regulation, DNA recombination and damage repair [33]. BCCIP suppresses the growth of certain tumor cells [34], but is required for tumor progression [35]. CD44 is a transmembranous glycoprotein which was involved in cell adhesion, cell migration, and metastasis [36, 37]. The BRG-1 subunit of the SWI/SNF complexes is a critical regulator of CD44 expression [38]. FLNB is one of the three isoforms of filamins which were actin-binding cross linking proteins [39]. FLNs were involved in initiation of cell migration [40]. These protein profiles help to explain how CAPZB exerts its tumor-accelerating effects in EpiS tissues.

In addition, it is interesting to note that the network analyses performed after the proteomics study identified several SWI/SNF chromatin-remodeling complexes including INI1 as upstream regulators of CAPZB, although INI1 itself was not included in the differentially expressed protein list according to CAPZB knockdown. INI1 is a key member of the SWI/SNF complex, and SWI/SNF complexes play essential roles in a variety of cellular processes, including differentiation, proliferation and DNA repair. The loss of SWI/SNF subunits has been reported in a number of malignant rhabdoid cell lines and tumors, and a large number of experimental observations suggest that this complex functions as a tumor suppressor [41]. In particular, loss of the INI1 protein expression has recently been reported in other tumors as well, including most cases of EpiS [42]. These findings prompted us to investigate the possible relationship between CAPZB and INI1 in EpiS. Consequently, silencing of CAPZB definitively suppressed cell proliferation in the setting of EpiS, although, surprisingly, the expression levels of INI1, which possesses a tumor suppressor function, were decreased in the ESX cells. On the other hand, the induction of INI1 in the INI1-negative EpiS cells (VAESBJ cells) definitively suppressed cell growth. Furthermore, according to INI1 induction, the CAPZB mRNA expression levels increased significantly (Fig. 4c), although the changes in the protein expression levels were not detectable by WB assays. These results suggest that the relationship between INI1 and CAPZB is complex and involves several co-mediators. We believe that further functional studies may help to clarify the interactions and networks between CAPZB and INI1 and that our functional and global protein expression data provide novel information for conducting such studies.

## Conclusions

In summary, we found that CAPZB contributes to the cell growth and motility of EpiS cells, irrespective of the INI1 expression, highlighting a possible role of CAPZB in metastasis and tumor development in cases of EpiS. Nevertheless, the paradoxical relationship between the tumor suppressor INI1 and the oncoprotein CAPZB in EpiS remains to be clarified.

## Availability of data and materials

The datasets supporting the conclusions of this article are included within the article and its additional files.

## Additional files

Additional file 1: Figure S1. Localization and expression of CAPZB in remaining fourteen cases of EpiS. (JPEG 1721 kb) Additional file 2: Figure S2. Localization and expression of CAPZB in remaining fourteen cases of EpiS. (JPEG 1323 kb) Additional file 3: Table S1. Results of the network analyses using protein profiles of CAPZB-regulated proteins in the VAESBJ cells. (XLSX 39 kb) Additional file 4: Table S2. Results of the network analyses using protein profiles of CAPZB-regulated proteins in the ESX cells. (XLSX 44 kb)

## Abbreviations

CAPZB: F-actin capping protein subunit beta
EpiS: Epithelioid sarcoma
i-TRAQ: Isobaric tags for relative and absolute quantitation
IPA: Ingenuity pathways analysis
SMARCB1: SWI/SNF-related matrix-associated actin-dependent regulator of chromatin subfamily B member 1
Cp: Capping protein
FFPE: Formalin-fixed, paraffin-embedded
IHC: Immunohistochemistry
LC-MS/MS: Liquid chromatography-tandem mass spectrometry
PCR: Polymerase chain reaction
CAPZA1: F-actin-capping protein subunit alpha-1
MX1: Interferon-induced GTP-binding protein MX1
BCCIP: BRCA2 and CDKN1A-interacting protein
SWI/SNF: Switch/sucrose non-fermentable
FLNB: Filamin B, beta
