# Supplementary figures and images for: Expression of F-actin-capping protein subunit beta, CAPZB, is associated with cell growth and motility in epithelioid sarcoma
Source: BMC Cancer. 2016 Mar 10;16:206. doi: 10.1186/s12885-016-2235-z (PMC4787035; doi:10.1186/s12885-016-2235-z)

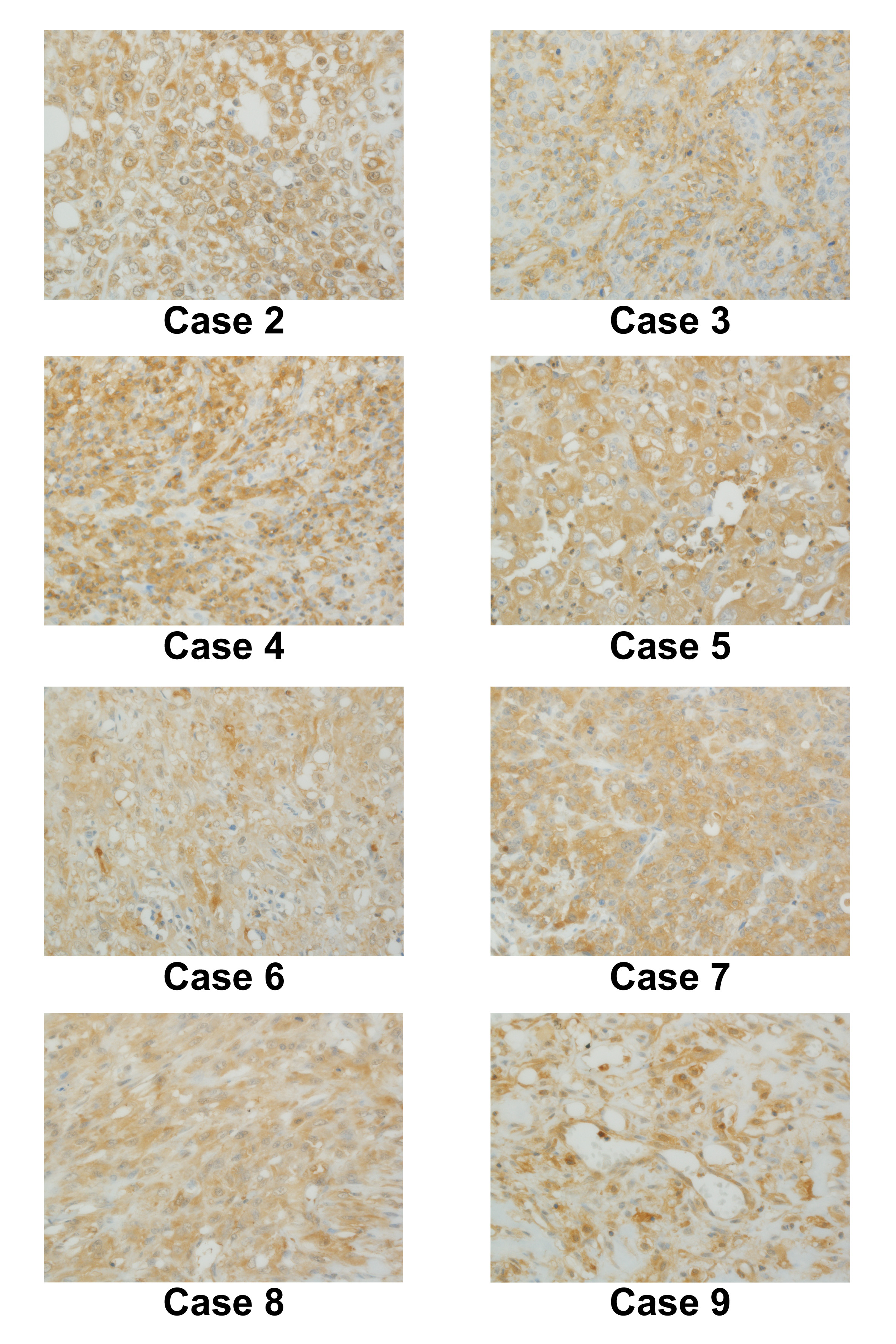

Supplement: Additional file 1: Figure S1. — Localization and expression of CAPZB in remaining fourteen cases of EpiS. (JPEG 1721 kb) [file 12885_2016_2235_MOESM1_ESM.jpeg]

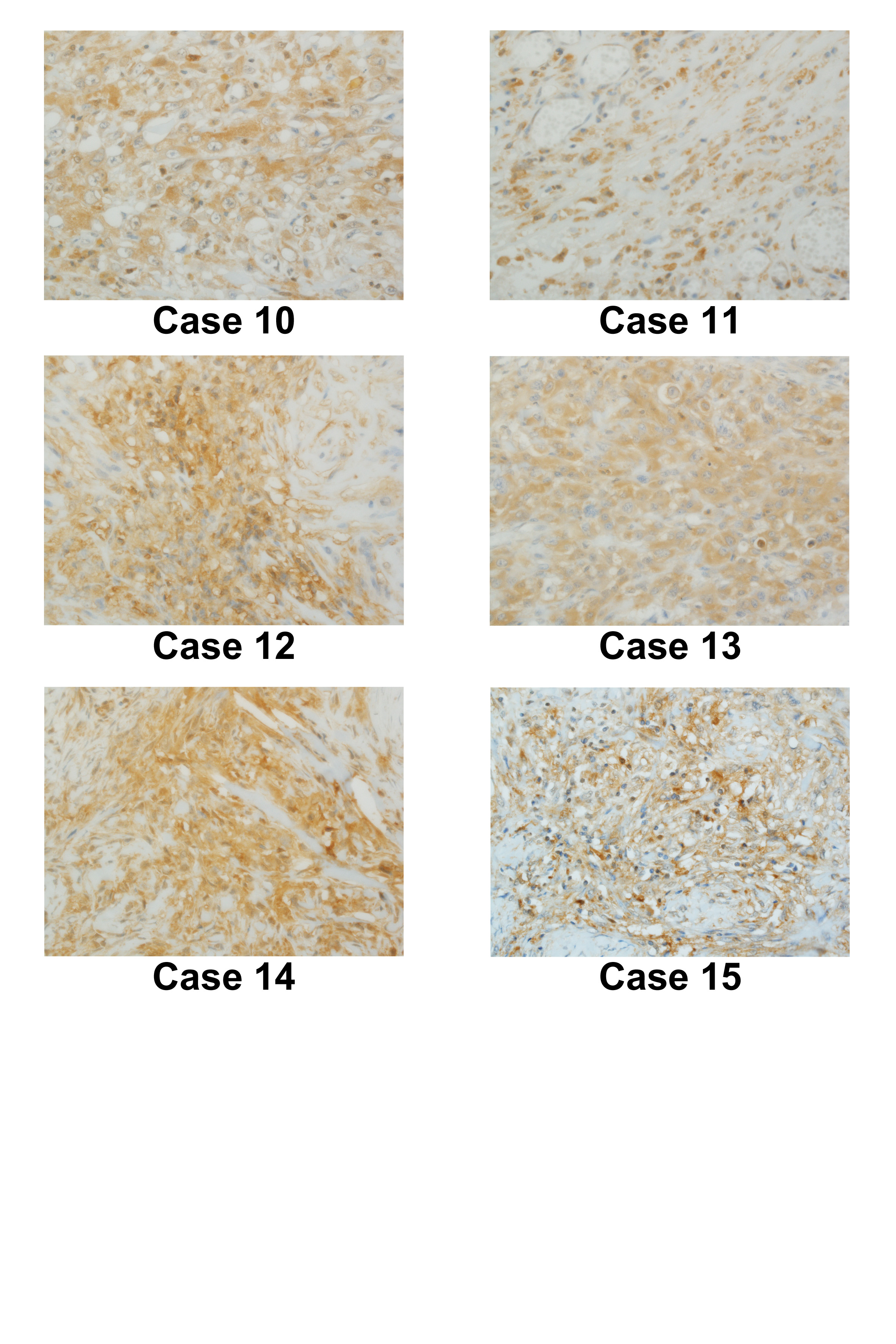

Supplement: Additional file 2: Figure S2. — Localization and expression of CAPZB in remaining fourteen cases of EpiS. (JPEG 1323 kb) [file 12885_2016_2235_MOESM2_ESM.jpeg]
